# Temporal Quantum-like Cognition in a Free-Energy Variational Framework

**DOI:** 10.3390/e28080848

**Published:** 2026-07-29

**Authors:** Shigenori Tanaka

**Affiliations:** Center for Life Photonic Innovation, Kobe University, 1-1 Rokkodai, Nada-ku, Kobe 657-8501, Japan; tanaka2@kobe-u.ac.jp

**Keywords:** cognition, decision making, consciousness, free energy principle, Schrödinger bridge, quantum state over time

## Abstract

Free-energy-based variational principles provide a powerful framework for describing perception, inference, and decision making. The Free Energy Principle (FEP) is one prominent realization of this idea, but its standard formulation is usually expressed in terms of classical probabilistic generative models. Here, we propose an FEP-inspired, quantum-like variational approach by integrating Quantum State Over Time (QSOT), Schrödinger-bridge (SB) theory, and quantum logic into a unified framework. Cognitive evolution is formulated as the optimization of temporally extended quantum-like states under a composite action consisting of an SB-inspired path divergence, a cognitive free-energy functional, and context-dependent quantum-logical constraints with terminal target propositions. Contextual updating is modeled by time-dependent non-selective Lüders projections, while endogenous cognitive steering is represented by an internal control field. A minimal two-state toy model illustrates how these components jointly shape temporal quantum-like cognitive trajectories. The proposed framework provides a temporally extended quantum-like analogue of the variational structure underlying the FEP and suggests a general mathematical basis for modeling context-dependent cognitive dynamics.

## 1. Introduction

Understanding how living systems perceive, infer, decide, and act remains one of the central challenges in contemporary science. Among the theoretical frameworks proposed to address this problem, the Free Energy Principle (FEP), introduced by Friston [1,2], has emerged as one of the most influential approaches for describing adaptive behavior in biological and cognitive systems. According to the FEP, cognitive agents continuously update their internal states so as to minimize a variational free energy functional, thereby reducing uncertainty about the external world and maintaining their existence within viable states. The framework has been successfully applied to perception, learning, decision making, active inference, and even consciousness.

Despite its broad applicability, the conventional formulation of the FEP is fundamentally classical. Internal beliefs are typically represented by classical probability distributions, and inference is described through Bayesian updating within a classical probabilistic framework. However, a growing body of evidence accumulated over the past two decades suggests that many aspects of human cognition and decision making are not always adequately described by classical probability theory. Phenomena such as order effects, conjunction and disjunction fallacies, violations of the sure-thing principle, and contextual dependence have motivated the development of quantum cognition, in which cognitive states are represented by vectors or density operators in Hilbert space and decisions are modeled as quantum measurements [3,4,5,6,7,8,9,10,11,12].

Quantum cognitive models have demonstrated remarkable success in reproducing experimentally observed deviations from classical rationality. In these models, interference, superposition, contextuality, and non-commutativity naturally emerge as key mechanisms underlying human judgment and decision making. Consequently, if quantum probabilistic descriptions provide a more faithful representation of cognitive processes, it becomes natural to reconsider the FEP within a quantum-theoretical framework.

In the present manuscript, the term “cognitive” is used in an operational modeling sense. A state, observable, projector, or Hamiltonian is called cognitive when it is defined on a state space whose basis elements represent cognitive alternatives, and when its operational role is specified in terms of recognizable cognitive functions such as judgment, belief revision, decision formation, attentional orientation, or action tendency. In this sense, the adjective “cognitive” does not refer to an intrinsic mathematical property of the operator alone, but to the semantic interpretation of the state space and to the functional role played by the operator in the model. In particular, the cognitive preference Hamiltonian introduced below is called cognitive because it defines a relative stability or preference structure over cognitive alternatives and generates controlled transitions among them. Thus, the present framework is intended from the outset as a quantum-like representation of temporally extended cognitive dynamics, rather than as an abstract formalism with only a possible future application to cognition.

Motivated by these perspectives, we recently proposed a quantum dynamical model of decision making based on the theory of open quantum systems [13]. In that study, the Prisoner’s Dilemma (PD) game [14] was used as a prototype example. The cognitive state of each player was represented by a density matrix, while a Hamiltonian was constructed such that the diagonal elements corresponded to expected utilities and the off-diagonal elements encoded quantum interference effects. The time evolution of the cognitive state was then obtained by solving the Liouville–von Neumann equation for a dissipative quantum system interacting with an environment characterized by an effective temperature. From the resulting density matrix, thermodynamic quantities including the von Neumann entropy, internal energy, and Helmholtz free energy were evaluated.

The numerical results revealed several characteristic quantum effects [13]. In particular, quantum interference produced deviations from classical decision-theoretic predictions, while the free energy decreased monotonically toward equilibrium during the decision process. A notable observation was that the nonequilibrium free-energy difference could be expressed as a quantum relative entropy (QRE) between the evolving density matrix (ρ(t)) and an equilibrium reference state ($\rho_{eq}$). Specifically,

(1) $$F\left(t\right)-F_{\mathrm{eq}}=\frac{1}{\beta }D_{QRE}\left(\rho \left(t\right)|\rho_{\mathrm{eq}}\right)\geq 0,$$

where $D_{QRE}\left(\rho |\sigma \right)$ denotes the quantum relative entropy and β is the inverse temperature. This relation represents a natural quantum extension of the variational free energy appearing in the classical FEP. It suggests that free-energy minimization in cognitive dynamics can be interpreted as an information-geometric process occurring in the space of quantum states (see also Appendix A).

Another recent development motivating the present work concerns the role of quantum measurement in cognition. Experimental and theoretical studies have suggested that repeated cognitive judgments may induce effects analogous to the quantum Zeno effect [15,16,17]. From a psychological perspective, this phenomenon may be interpreted as a form of belief fixation or cognitive rigidity, in which excessive repetition of judgments suppresses transitions between alternative mental states. Such effects provide a possible quantum-mechanical description of confirmation bias and other forms of constrained cognition. While these studies highlight the importance of measurement-induced dynamics [11], they also reveal a limitation of conventional quantum cognition models, where cognitive states are typically defined only at a single instant of time.

Human cognition, however, is inherently temporal. Perception, memory, anticipation, planning, and conscious experience are not confined to the present moment but continuously integrate information from the past while generating expectations about the future. Consciousness therefore appears to possess an intrinsically time-extended structure. Capturing such temporal organization requires a framework that goes beyond instantaneous quantum states. To address this issue, we here adopt the recently developed formalism of Quantum State Over Time (QSOT) [18,19]. The QSOT framework extends the standard notion of quantum states by representing correlations across multiple times within a unified mathematical object. In this way, temporal relationships can be treated analogously to spatial correlations in conventional quantum theory. The framework provides a natural language for describing memory effects, temporal coherence, and history-dependent cognitive processes.

In parallel, another line of development has emerged from Schrödinger’s original bridge problem [20], which has recently attracted renewed attention [21] in optimal transport theory, stochastic control, and machine learning. The Schrödinger bridge (SB) formalism describes the most probable stochastic evolution connecting prescribed initial and final probability distributions under an entropy-minimization principle. Because both the FEP and SB theory are fundamentally variational in nature, a deep conceptual relationship exists between them. Integrating these ideas within a quantum framework offers a promising route toward a unified description of cognition as an entropy-constrained dynamical inference process.

The purpose of the present work is therefore to develop a new theoretical framework for quantum cognition and decision making that combines three complementary concepts: (i) a quantum-logical, free-energy-based variational approach inspired by the FEP, (ii) the temporal representation provided by QSOT, and (iii) the variational structure of the SB problem. Within this framework, cognitive states are represented as temporally extended quantum objects whose evolution is governed by free-energy minimization under quantum-mechanical constraints. We formulate the resulting theory, derive its fundamental variational structure, and illustrate its behavior through model calculations. The proposed approach, which is an extension of preceding work [22], provides a unified perspective on cognition, decision making, and consciousness as dynamical processes occurring in a temporally extended quantum-information space.

We hypothesize that consciousness is fundamentally a temporally extended quantum-informational process governed by a principle of free-energy minimization. In this view, conscious experience is represented by a QSOT, which integrates past memories, present perceptions, and future anticipations into a unified cognitive state. The evolution of this state is driven by the minimization of quantum variational free energy, while quantum measurements and environmental interactions continuously update its temporal structure. Consequently, cognition, decision making, and consciousness can be regarded as different manifestations of a single underlying process: the optimal evolution of temporally extended quantum states under informational and thermodynamic constraints.

This study introduces a minimal QSOT toy model in which a two-level cognitive state evolves under internal control, context-dependent logical projection and decoherence. The realized temporal cognitive trajectory is selected by minimizing an SB-type variational functional consisting of a path-space relative entropy, a thermodynamic free-energy term, and quantum-logical constraints imposed by contextual and goal projectors.

The remainder of this paper is organized as follows. Section 2 introduces the mathematical formulation of the temporal quantum cognitive framework. Section 3 and Section 4 present a minimal toy model and numerical simulations. Section 5 discusses the implications of model calculations in the light of the connections among quantum relative entropy, free-energy minimization, temporal correlations, and cognitive dynamics. Finally, Section 6 summarizes the main conclusions and outlines future directions for the development of quantum theories of cognition and consciousness.

## 2. Theory

In an earlier work [13] we employed a quantum dynamical model of open system to solve the quantum PD problem and calculate the time evolution of the density matrix ρ(t) of the player system, which represents the cognitive/belief or decision state of players. Once ρ(t) is found, the entropy *S*, internal energy *E*, and free energy *F* of the system can be calculated as follows:

(2) F(t)=E(t)−TS(t),

(3) $$E\left(t\right)=\mathrm{Tr}\left[\rho \left(t\right)H_{S}\right],$$

(4) $$S\left(t\right)=-\mathrm{Tr}\left[\rho (t)\log\rho (t)\right].$$

Here, $H_{S}$ is the system Hamiltonian, and the entropy *S* denotes the von Neumann entropy of the cognitive density operator. In the present framework this quantity characterizes the uncertainty (or mixedness) of the cognitive quantum state and is not assumed *a priori* to coincide with phenomenological thermodynamic entropy. As time t→∞, the system approaches the thermal equilibrium at temperature *T* represented by the equilibrium density matrix as

(5) $$\rho_{\mathrm{eq}}=\frac{e^{-\beta H_{S}}}{\mathrm{Tr}\left(e^{-\beta H_{S}}\right)}$$

with the inverse temperature β=1/T. Using Klein’s inequality, one can show that there is a relationship between the equilibrium free energy $F_{\mathrm{eq}}$ and the free energy *F* at any time *t* as

(6) $$\beta F-\beta F_{\mathrm{eq}}=\mathrm{Tr}\left[\rho (\log\rho -\log\rho_{\mathrm{eq}})\right]\geq \mathrm{Tr}\left(\rho -\rho_{\mathrm{eq}}\right)=0,$$

indicating that $F_{\mathrm{eq}}$ gives a lower bound. This model provides a prior basis for the present study. (See Appendix A for more details.)

Here, to express a temporally correlated cognitive/conscious state, we consider a quantum state over time (QSOT), which represents a continuous set of time-dependent density matrix $\rho_{t}$ defined over a temporal domain between t=0 and t=T:

(7) $$\Omega_{0:T}={\left\{\rho_{t}\right\}}_{0\leq t\leq T}.$$

We note that cognition is not a static state but a temporally constrained trajectory.

We look for a variational solution of QSOT (briefly referred to as Ω afterwards) which is the nearest to a reference cognitive path *Q* along with the minimal free energy F[Ω] and some constraints $C_{k}\left[\Omega \right]$ associated with the contextual relevance of cognition:

(8) $$\Omega^{*}=\arg\min_{\Omega }\left[\lambda_{\mathrm{KL}}D_{\mathrm{KL}}\left(\Omega ||Q\right)+\lambda_{F}F\left[\Omega \right]+\sum_{k}\lambda_{k}C_{k}\left[\Omega \right]\right].$$

Here, $D_{\mathrm{KL}}$ means the (generalized) Kullback–Leibler (KL) divergence or the quantum relative entropy (QRE) with the weight $\lambda_{\mathrm{KL}}$, which requires that the optimal QSOT ($\Omega^{*}$) should not deviate from a given path *Q* in the sense of Schrödinger bridge (SB) [20,21].

The free energy with the weight $\lambda_{F}$, which represents a cognitive stability or preference, is given as

(9) $$F\left(\rho_{t}\right)=E\left(\rho_{t}\right)-T_{\mathrm{cog}}S\left(\rho_{t}\right),$$

(10) $$E\left(\rho_{t}\right)=\mathrm{Tr}\left(\rho_{t}H_{\mathrm{cog}}\right),$$

(11) $$S\left(\rho_{t}\right)=-\mathrm{Tr}\left(\rho_{t}\log\rho_{t}\right),$$

(12) $$F\left[\Omega \right]=\int_{0}^{T}dtF\left(\rho_{t}\right)$$

with the introduction of cognitive preference Hamiltonian $H_{\mathrm{cog}}$, which defines a stability or preference operator over cognitive alternatives in the operational sense mentioned above, and associated cognitive temperature $T_{\mathrm{cog}}$. Here, $F(\rho_{t})$ should be regarded as a cognitive free-energy functional at time *t* that combines energetic preference and information-theoretic uncertainty, rather than as the thermodynamic Helmholtz free energy of a physical equilibrium system.

The contextuality (logical consistency) constraints $C_{k}\left[\Omega \right]$ on QSOT with the weights $\lambda_{k}$ can be expressed as

(13) $$\sum_{k}\lambda_{k}C_{k}\left[\Omega \right]=\lambda_{\mathrm{ctx}}C_{\mathrm{ctx}}\left[\Omega \right]+\lambda_{T}C_{T}\left[\Omega \right],$$

(14) $$C_{\mathrm{ctx}}\left[\Omega \right]=\int_{0}^{T}dt{\left(\mathrm{Tr}\left(\rho_{t}\Pi_{\mathrm{ctx}}\left(t\right)\right)-m_{\mathrm{ctx}}\left(t\right)\right)}^{2},$$

(15) $$C_{T}\left[\Omega \right]={\left(\mathrm{Tr}\left(\rho_{T}\Pi_{T}\right)-m_{T}\right)}^{2},$$

where $\Pi_{\mathrm{ctx}}\left(t\right)=\left|\phi \left(t\right)\rangle \langle \phi \left(t\right)\right|$ and $\Pi_{T}$ (for goal at t=T) refer to the projection operators onto given (contextual) quantum states, and $m_{\mathrm{ctx}}\left(t\right)$ and $m_{T}$ are target values at times *t* and *T*, respectively.

Given an appropriate initial condition, the time-dependent density matrix obeys the equation of motion:

(16) $$\frac{d\rho_{t}}{dt}=-i\left[H\left(t\right),\rho_{t}\right]+L_{\mathrm{ctx}}\left[\rho_{t}\right]+L_{\mathrm{noise}}\left[\rho_{t}\right],$$

where H(t) refers to a time-dependent Hamiltonian specified later, and $L_{\mathrm{ctx}}\left[\rho_{t}\right]$ and $L_{\mathrm{noise}}\left[\rho_{t}\right]$ are the Lindblad-type relaxation operators representing the contextual projection (context-dependent logical updating by weak quantum measurement) and noise (decoherence/dephasing or forgetting) effects, respectively (see the next section). Thus, in contrast to Friston’s FEP for belief update [1,2], the present theory aims to describe a trajectory optimization over temporal quantum logic in the framework of SB along with free energy minimization and contextual selection.

## 3. Toy Model

On the basis of formal theory presented in the preceding section, we perform a specific toy model calculation in this section. Let us consider a two-state model, in which $|0\rangle ={\left(1,0\right)}^{T}$ and $|1\rangle ={\left(0,1\right)}^{T}$ represent two abstract cognitive alternatives, such as competing beliefs, decisions, hypotheses, or action tendencies. For the time-dependent Hamiltonian introduced in Equation (16), we consider

(17) $$H\left(u,t\right)=H_{\mathrm{cog}}+u\left(t\right)H_{\mathrm{ctrl}},$$

(18) $$H_{\mathrm{cog}}=\frac{\Delta }{2}\sigma_{z},$$

(19) $$H_{\mathrm{ctrl}}=\frac{1}{2}\sigma_{y},$$

(20) $$\sigma_{z}=\left(\begin{array}{cc} 1 & 0\\ 0 & -1 \end{array}\right),\;\sigma_{y}=\left(\begin{array}{cc} 0 & -i\\ i & 0 \end{array}\right),$$

where $H_{\mathrm{cog}}$ is the cognitive preference Hamiltonian introduced in Equation (10), representing that there is a difference Δ in psychological stability between the two states. The second term in Equation (17) refers to the contribution by internal cognitive steering (or active inference) u(t) for rotating the quantum state on the Bloch sphere.

We then transform the variational problem with respect to Ω to that of u(t): $\Omega^{*}=\Omega \left[u^{*}\right]$. The optimal u(t) is obtained through

(21) $$u^{*}=\arg\min_{u}A\left[\Omega [u]\right]$$

with a functional:

(22) $$A\left[\Omega [u]\right]=\int_{0}^{T}\frac{1}{2}u{\left(t\right)}^{2}dt+\lambda_{\mathrm{KL}}\int_{0}^{T}D_{\mathrm{QRE}}(\rho_{t}^{\Omega }\left|\right|\rho_{t}^{Q})dt+\lambda_{F}F\left[\Omega \right]+\lambda_{T}C_{T}\left[\Omega \right]+\lambda_{\mathrm{ctx}}C_{\mathrm{ctx}}\left[\Omega \right].$$

Here, the second term in Equation (22) refers to the difference in the density matrices between those calculated with and without u(t). In the present proof-of-concept implementation, this Schrödinger bridge (SB) term is represented by a path-integrated quantum relative entropy (QRE) between instantaneous states:

(23) $$D_{\mathrm{KL}}\left(\Omega ||Q\right)≃\int_{0}^{T}D_{\mathrm{QRE}}(\rho_{t}^{\Omega }\left|\right|\rho_{t}^{Q})dt.$$

This path-integrated QRE approximation should be regarded as a surrogate of the true path-space divergence between Ω and *Q*. A more rigorous formulation would require the construction of quantum path measures and their associated relative entropy, which remains an important direction for future work.

The first term in Equation (22) denotes the penalty (cognitive cost) associated with the introduction of u(t). The control field u(t) is not externally prescribed. It is determined variationally as the minimum-effort temporal modulation that generates a controlled trajectory Ω[u]. The optimized trajectory $\Omega^{*}=\Omega \left[u^{*}\right]$ is selected as the minimally deformed trajectory relative to the uncontrolled reference process *Q*, while satisfying terminal and contextual constraints together with free-energy minimization. The present study thus provides an SB-like minimum-action temporal inference model.

The time evolution of the density matrix was computed using a first-order operator-splitting scheme on the basis of Equation (16). For each time step (Δt), the evolution was decomposed into three successive operations: (i) contextual logical projection, (ii) unitary evolution generated by the cognitive Hamiltonian, and (iii) environmental decoherence. The procedure was repeated sequentially over the entire time interval. The resulting algorithm preserves Hermiticity, positivity, and trace normalization of the density matrix throughout the simulation.

As for the relaxation operators in Equation (16), we employed

(24) $$L_{\mathrm{ctx}}\left[\rho \right]=\eta \left(\Pi \rho \Pi +\Pi^{⊥}\rho \Pi^{⊥}-\rho \right)$$

with $\Pi =\Pi_{\mathrm{ctx}}\left(t\right)$ and $\Pi^{⊥}=I-\Pi$ for the contextual projection (framing) characterized by a small (weak amplitude) parameter η, which partially suppresses the quantum coherence without fully collapsing the state. The contextual projection operator $\Pi_{\mathrm{ctx}}\left(t\right)=\left|\phi \left(t\right)\rangle \langle \phi \left(t\right)\right|$ is described by |ϕ(t)〉=cosα(t)|0〉+sinα(t)|1〉 with the time-dependent cognition context α(t). For the noise term, we employed the decoherence operator as

(25) $$L_{\mathrm{noise}}\left[\rho \right]=\gamma \left(\sigma_{z}\rho \sigma_{z}-\rho \right)$$

with the relaxation rate γ. For simplicity, the present toy model employs a pure dephasing channel represented by a Pauli-$\sigma_{z}$ Lindblad operator. More general formulation can be obtained by replacing this term with an arbitrary Lindblad generator. In cognitive terms, this environmental contribution may be interpreted as background cognitive noise, forgetting, uncontrolled memory associations, or general knowledge context, in line with recent open-system approaches to quantum cognition [23].

In the description above, the contextual influence is modeled by a non-selective Lüders projection term, $L_{\mathrm{ctx}}\left[\rho \right]$, where $\Pi =\Pi_{\mathrm{ctx}}\left(t\right)$ denotes the contextual projector and $\Pi^{⊥}=I-\Pi$ its orthogonal complement. This term represents a context-dependent logical update that differs fundamentally from ordinary unitary evolution. Whereas the Hamiltonian part describes intentional cognitive steering, the Lüders term models the effect of exposing the cognitive state to a particular context. The context does not force the system into one specific outcome. Instead, it selectively suppresses coherence between the contextual subspaces defined by Π and $\Pi^{⊥}$. Mathematically, the operation $\Pi \rho \Pi +\Pi^{⊥}\rho \Pi^{⊥}$ refers to the standard non-selective Lüders transformation. It preserves the probabilities associated with the contextual alternatives while eliminating the off-diagonal interference terms between them. Consequently, the cognitive state becomes increasingly aligned with the logical structure induced by the current context. The parameter η controls the strength of contextual influence. For η=0, the context has no effect and the dynamics reduce to the controlled quantum evolution. As η increases, the cognitive state is progressively reorganized according to the contextual partition represented by Π and $\Pi^{⊥}$. From the perspective of quantum cognition, this process may be interpreted as a contextual re-framing of mental representations. A context does not merely reveal a pre-existing cognitive state; rather, it actively modifies the structure of possible judgments by changing the effective logical decomposition of the state space.

In the present theory, cognition is therefore described as a continuous interplay between intentional steering and context-induced logical projection. Importantly, the contextual projector Π is allowed to vary with time. The resulting sequence of non-selective Lüders updates generates a temporally evolving logical environment, providing a dynamical realization of context-dependent cognition within the QSOT framework. Within the present framework, the non-selective Lüders term plays a role analogous to Bayesian conditioning in classical inference [11]. However, the update acts on an orthomodular quantum-logical structure rather than on a Boolean probability space. The contextual projector therefore defines a temporary logical perspective from which the cognitive state is evaluated. Cognition is consequently viewed not as inference within a fixed logical framework, but as a process in which the logical framework itself evolves over time through context-dependent projections.

Finally, to clarify the cognitive interpretation of the present formalism, we summarize the intended correspondence between the mathematical objects and psychological concepts. The density matrix $\rho_{t}$ represents the cognitive state of an agent at time *t*, encoding both uncertainty among alternative propositions and possible interference between them. In the present two-state example, the basis states |0〉 and |1〉 should not be interpreted as physical quantum states, but as abstract cognitive alternatives, such as two competing beliefs, decisions, hypotheses, or action tendencies. In addition, $H_{\mathrm{cog}}$ is not a physical brain Hamiltonian, but describes a relative stability between the two psychological states. The contextual projector $\Pi_{\mathrm{ctx}}\left(t\right)$ represents the currently operative interpretive frame. Its time dependence expresses the fact that the cognitive relevance of propositions may change during deliberation. The non-selective Lüders update induced by $\Pi_{\mathrm{ctx}}\left(t\right)$ models contextual reframing; the context does not force a single outcome, but reorganizes the cognitive state by suppressing interference between context-defined alternatives. The terminal projector $\Pi_{T}$ represents a future-oriented target proposition, such as a decision, belief commitment, or intended action. The control field u(t) represents endogenous cognitive steering, including attention, intention, or active reorientation of the state toward a future proposition. The reference process *Q* refers to the uncontrolled or spontaneous cognitive trajectory that would occur in the absence of such active steering. The optimized trajectory $\Omega^{*}$ is therefore interpreted as the minimally modified cognitive path that satisfies contextual and terminal constraints while remaining close to the spontaneous dynamics.

## 4. Numerical Results

The numerical calculation for 0≤t≤T=10.0 was carried out using the following conditions and parameters. The initial condition for the quantum state is $|\psi_{0}\rangle =\cos\theta_{0}|0\rangle +\sin\theta_{0}|1\rangle$ with $\theta_{0}=0.20$. The time-dependent cognition context is modeled as $\alpha \left(t\right)=\alpha_{0}+\left(\alpha_{1}-\alpha_{0}\right)t/T$ with $\alpha_{0}=0.15$ and $\alpha_{1}=1.25$. The contextuality parameter and the relaxation rate are chosen as η=0.035 and γ=0.05. In the functional *A* of Equation (22), the weight parameters are set to $\lambda_{\mathrm{KL}}=0.8$, $\lambda_{F}=0.35$, $\lambda_{T}=100.0$ and $\lambda_{\mathrm{ctx}}=50.0$. The cognition temperature is $T_{\mathrm{cog}}=0.20$, the energy difference between the two states is Δ=1.0, and the contexual targets are given by $m_{\mathrm{ctx}}\left(t\right)=0.55+0.25\sin\left(\pi t/T\right)$ and $m_{T}=0.90$ with $\Pi_{T}=\left|1\rangle \langle 1\right|$. The temporal domain was divided by N=100 so that the time step was Δt=T/N=0.1.

In the present formulation, the primary variational object is the temporally extended cognitive state $\Omega_{0:T}$. However, since the temporal trajectory is dynamically generated by the control field u(t), the variational problem may equivalently be formulated as an optimal-control problem over admissible temporal controls. Numerically, u(t) was represented as a piecewise-constant control vector $u=(u_{0},\ldots ,u_{N-1})$ on the discretized time grid with N=100. For each trial *u*, the trajectory Ω[u] was generated by the operator-splitting propagation of $\rho_{t}$, and the action A[Ω[u]] was evaluated. The finite-dimensional minimization was then performed using the L-BFGS-B algorithm, starting from $u_{n}=0$, with bounds $-2.5\leq u_{n}\leq 2.5$.

**Figure 1:**
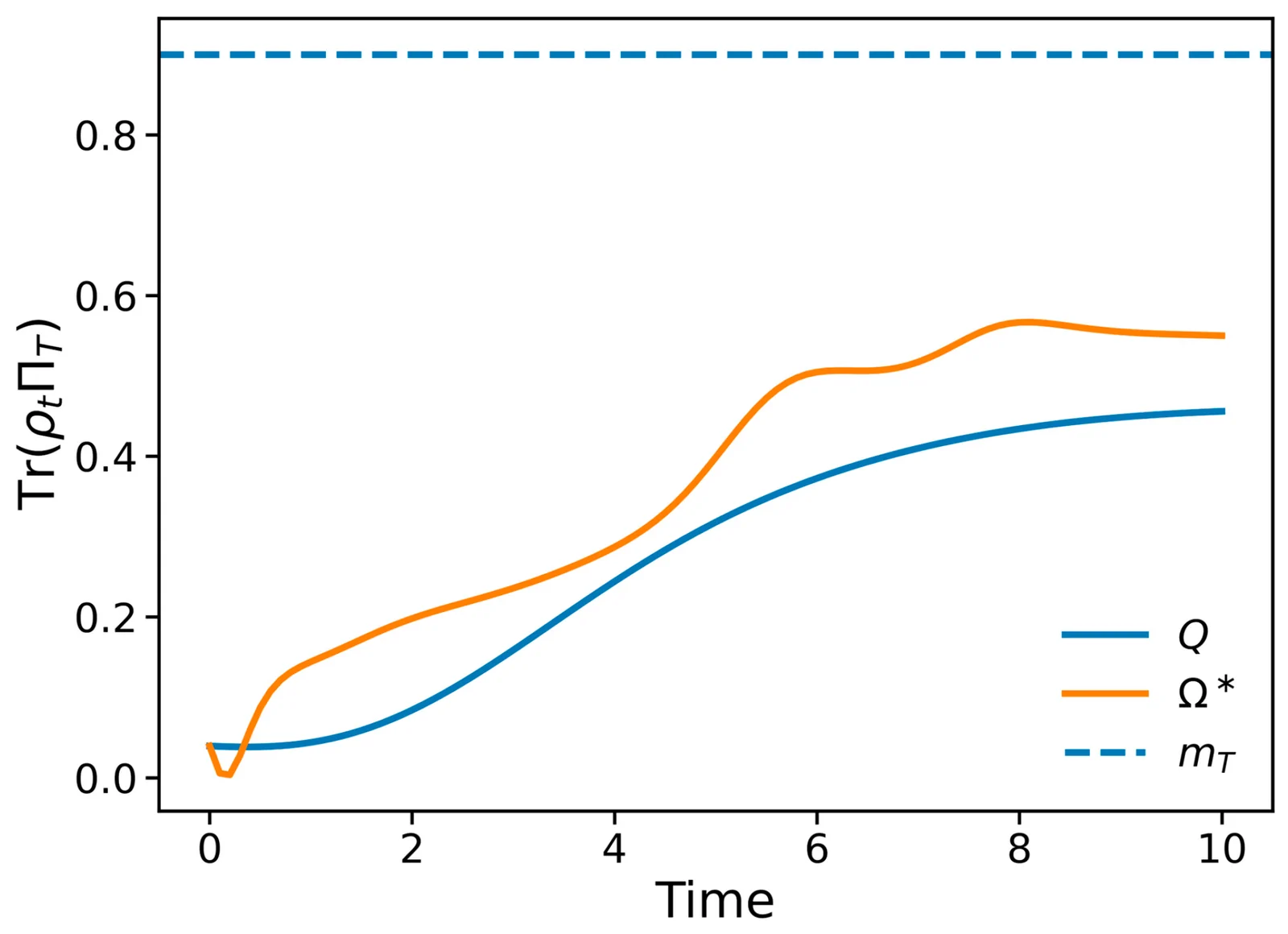
Temporal evolution of the terminal proposition weight, $\mathrm{Tr}(\rho_{t}\Pi_{T})$, for the uncontrolled reference process *Q* (blue curve) and the optimized trajectory $\Omega^{*}$ (orange curve). The dashed horizontal line indicates the prescribed target proposition value ($m_{T}=0.90$).

Figure 1 shows the temporal evolution of the terminal proposition weight, $\mathrm{Tr}(\rho_{t}\Pi_{T})$, for the uncontrolled reference process (*Q*) and the optimized trajectory ($\Omega^{*}$). The optimized trajectory is obtained by minimizing the action functional *A* under the terminal proposition constraint ($m_{T}=0.90$). The dashed horizontal line indicates the prescribed target proposition value. While the uncontrolled process remains far from the target proposition, the optimized trajectory progressively (but slightly) approaches the desired terminal proposition value through cognitive control u(t) (0.456→0.550 at t=T=10.0). This figure illustrates how the variational principle steers the cognitive state toward a specified future proposition while remaining close to the reference process.

**Figure 2:**
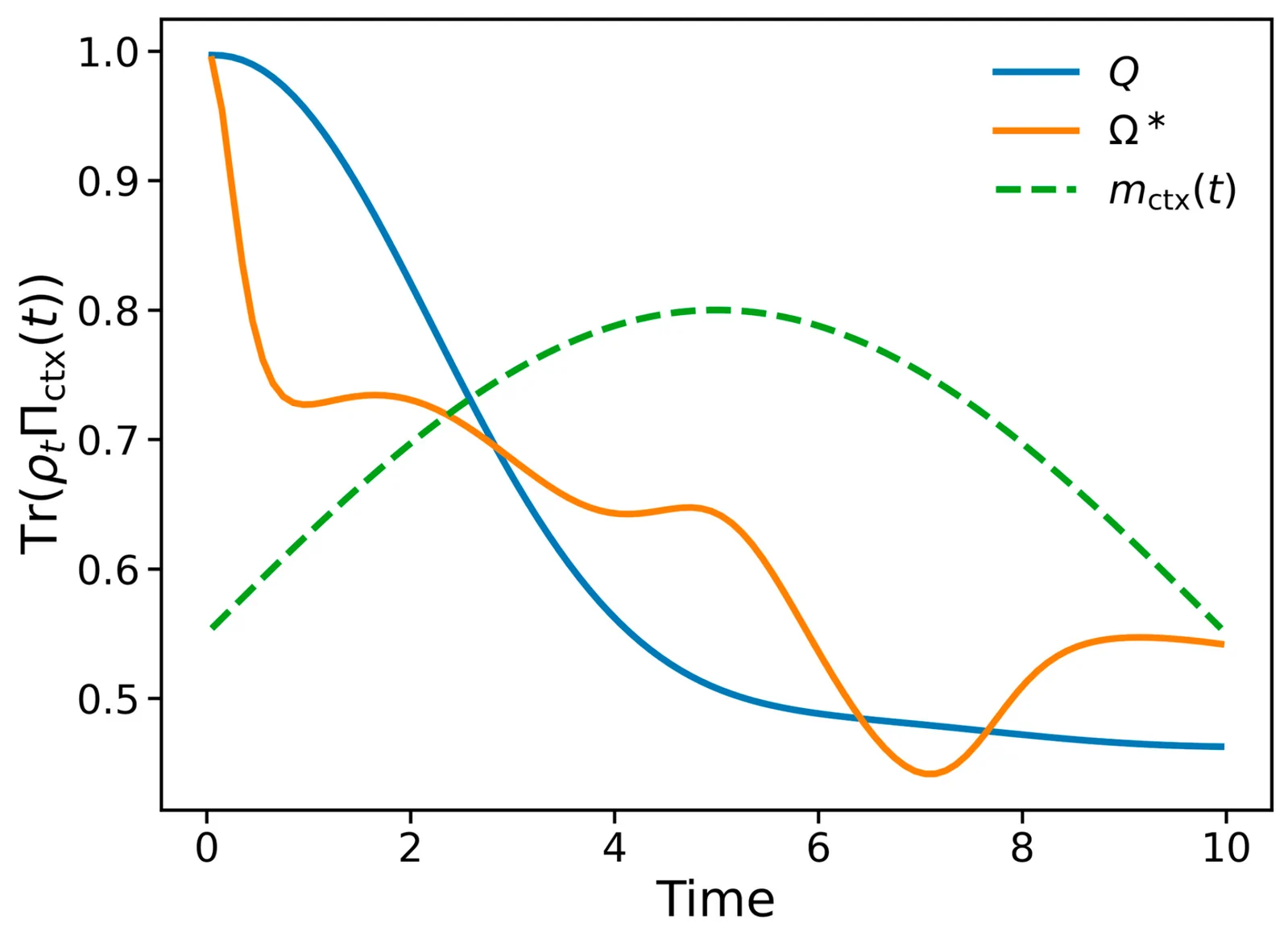
Temporal evolution of the contextual projection probability, $\mathrm{Tr}(\rho_{t}\Pi_{\mathrm{ctx}}\left(t\right))$, for the uncontrolled reference process *Q* (blue curve) and the optimized trajectory $\Omega^{*}$ (orange curve). The dashed green curve represents the prescribed contextual target function $m_{\mathrm{ctx}}\left(t\right)$.

Figure 2 illustrates the temporal evolution of the contextual projection probability, $\mathrm{Tr}(\rho_{t}\Pi_{\mathrm{ctx}}\left(t\right))$, for the uncontrolled reference process (*Q*) and the optimized trajectory ($\Omega^{*}$). The dashed curve represents the prescribed contextual target function $m_{\mathrm{ctx}}\left(t\right)$. The contextual projector $\Pi_{\mathrm{ctx}}\left(t\right)$ evolves continuously through the time-dependent context angle α(t), generating a dynamically changing logical environment. The optimized trajectory follows the contextual target more closely than the reference process, demonstrating how repeated non-selective Lüders updates reorganize the cognitive state in accordance with context-dependent quantum-logical constraints.

As seen in Figure 2, the optimized trajectory does not necessarily coincide with the prescribed contextual target profile $m_{\mathrm{ctx}}\left(t\right)$, because the contextual logical term enters the variational principle as a soft constraint competing with terminal goal attainment, thermodynamic free-energy minimization, and SB proximity to the reference process. The resulting trajectory therefore reflects a compromise among multiple cognitive objectives, implying cognitive frustration or conflicting intentions.

**Figure 3:**
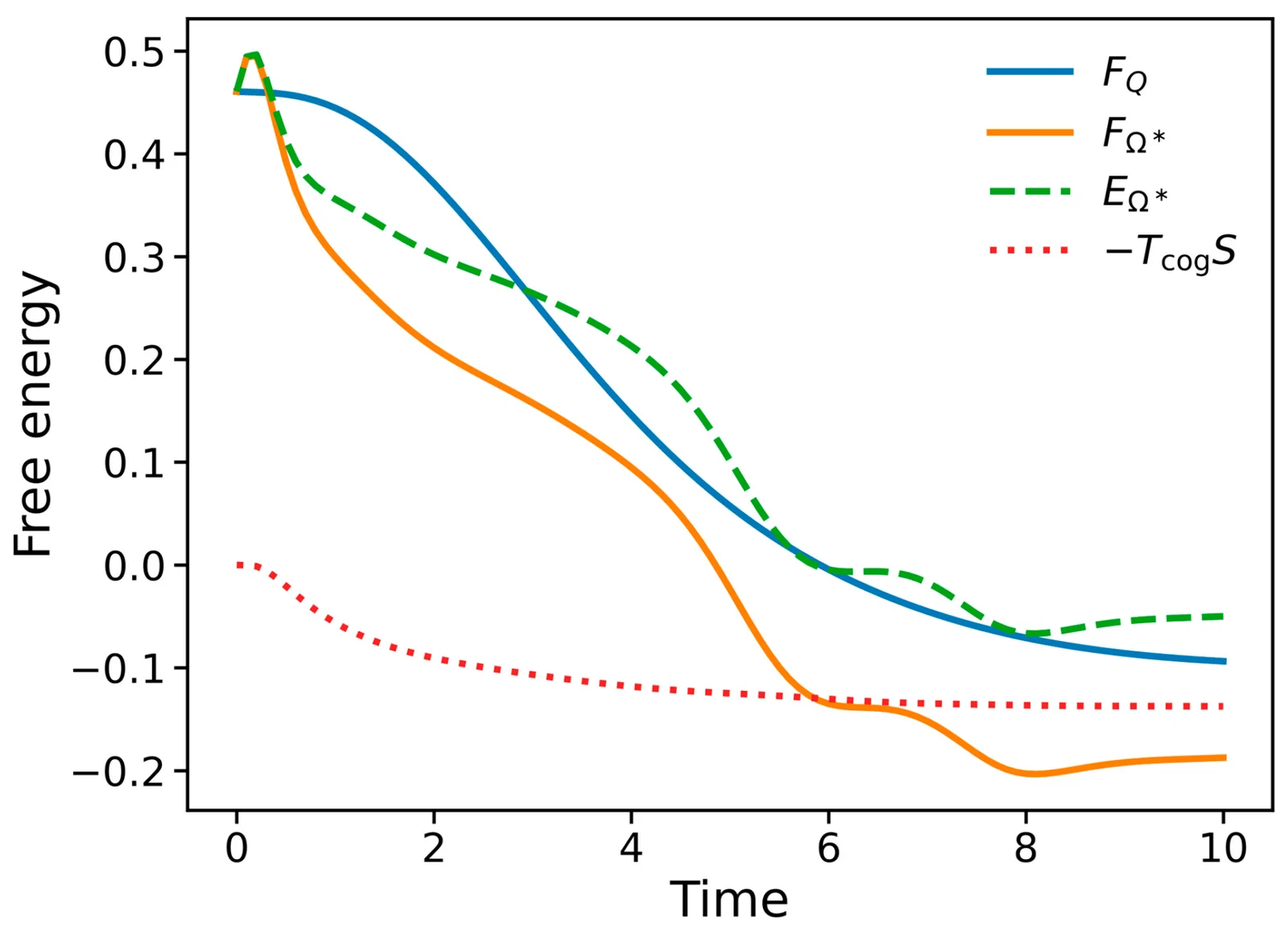
Free energy decomposition along the optimized cognitive trajectory. Orange, dashed green, and dotted red curves depict the free energy ($F_{\Omega^{*}}=E_{\Omega^{*}}-T_{\mathrm{cog}}S$), the corresponding internal cognitive energy ($E_{\Omega^{*}}$), and the entropic contribution ($-T_{\mathrm{cog}}S$), respectively. Blue curve illustrates the free energy of the uncontrolled reference process ($F_{Q}$). The cognitive temperature is $T_{\mathrm{cog}}=0.20$.

Figure 3 shows the thermodynamic free-energy decomposition along the optimized cognitive trajectory. The figure shows the free energy ($F_{\Omega^{*}}=E_{\Omega^{*}}-T_{\mathrm{cog}}S$), the corresponding internal cognitive energy ($E_{\Omega^{*}}$), and the entropic contribution ($-T_{\mathrm{cog}}S$), together with the free energy of the uncontrolled reference process ($F_{Q}$). The cognitive temperature was fixed at $T_{\mathrm{cog}}=0.20$. The optimized trajectory is selected not only by contextual and terminal logical constraints but also by minimizing the accumulated thermodynamic free energy. The figure illustrates how energetic and entropic contributions jointly determine the preferred cognitive evolution whose free energy is lower than $F_{Q}$. It is also noted that the resulting entropy *S* becomes positive, showing an emergence of disorder in the present case [24,25].

**Figure 4:**
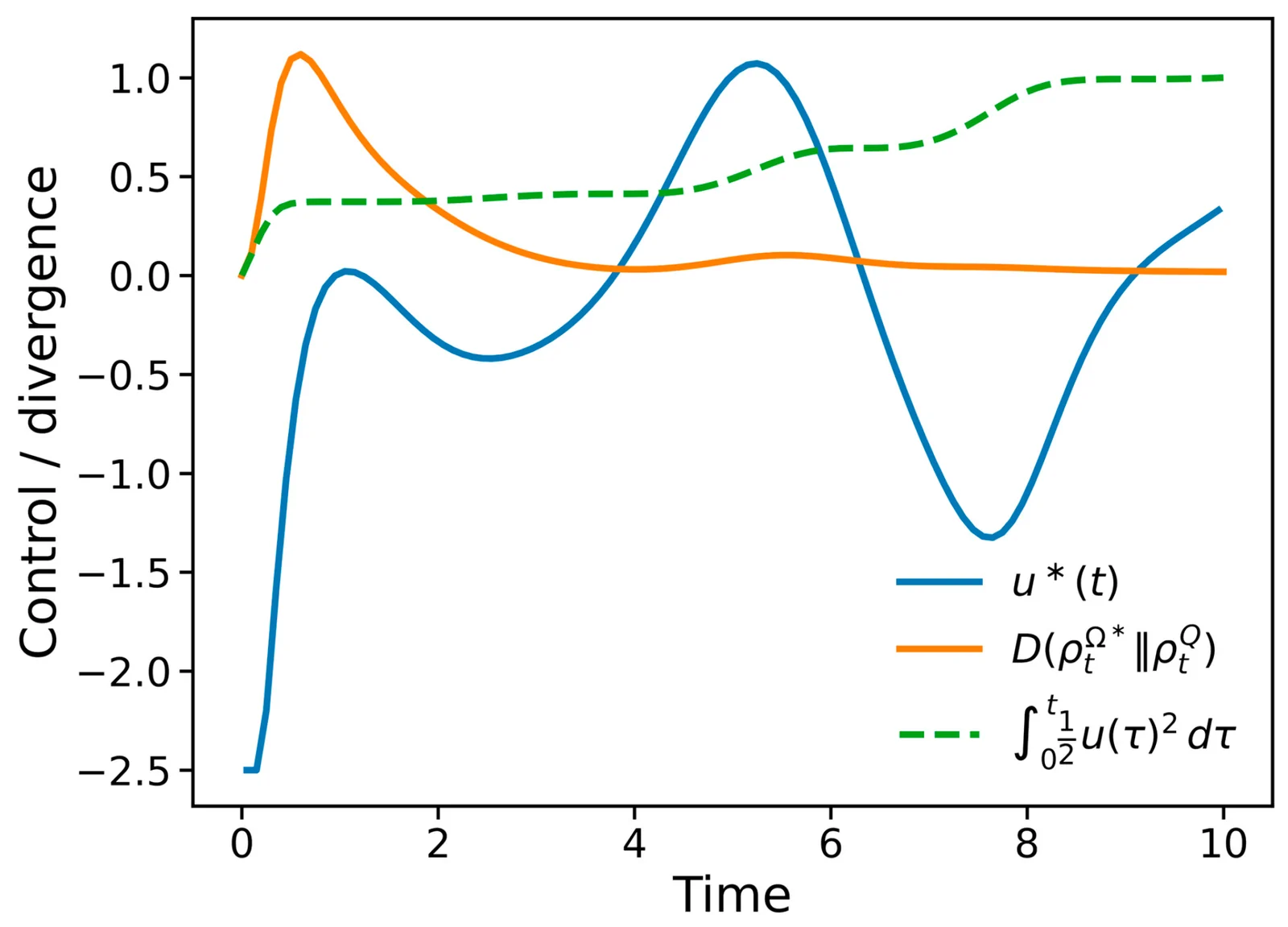
Schrödinger-bridge-like path cost. Blue solid curve shows the optimal cognitive control field $u^{*}\left(t\right)$, while orange solid curve represents the state-wise quantum relative entropy $D_{QRE}\left(\rho_{t}^{\Omega^{*}}∥\rho_{t}^{Q}\right)$ between the optimized trajectory and the uncontrolled reference process. Green dashed curve shows the normalized cumulative control action ($\int_{0}^{t}\frac{1}{2}u{\left(\tau \right)}^{2}d\tau$).

Figure 4 depicts the components of the SB-like path cost. The blue solid curve shows the optimal cognitive control field $u^{*}\left(t\right)$, while the orange solid curve represents the state-wise quantum relative entropy $D_{QRE}\left(\rho_{t}^{\Omega^{*}}∥\rho_{t}^{Q}\right)$ between the optimized trajectory and the uncontrolled reference process. The green dashed curve shows the normalized cumulative control action ($\int_{0}^{t}\frac{1}{2}u{\left(\tau \right)}^{2}d\tau$). Together, these quantities characterize the compromise underlying the optimized trajectory: the system seeks to satisfy contextual and terminal logical constraints while remaining close to the reference process and minimizing control expenditure.

The minimal model considered above assumes that the cognitive control field u(t) can vary arbitrarily in time, subject only to the energetic control cost $\int_{0}^{T}\left(1/2\right)u{\left(t\right)}^{2}dt$. Although this assumption is sufficient to demonstrate the basic mechanism of the proposed QSOT–SB–FEP framework, the resulting optimal control trajectories may exhibit relatively rapid temporal variations. From a cognitive perspective, such abrupt changes are not always realistic. Human attention, intention, and decision-making processes typically possess temporal persistence and cannot be reoriented instantaneously. To account for this effect, one may introduce an additional regularization term that penalizes rapid variations of the control field such as $\int_{0}^{T}{\left(\frac{du}{dt}\right)}^{2}dt$. The variational functional is therefore extended to $A+\lambda_{\dot{u}}\int_{0}^{T}{\left(\frac{du}{dt}\right)}^{2}dt$, where $\lambda_{\dot{u}}$ controls the degree of temporal smoothness. Mathematically, this term imposes a Sobolev-type regularity condition on the control trajectory. In optimal-control terminology, it penalizes rapid changes of the steering field. From the viewpoint of cognition, it may be interpreted as a temporal inertia of attention or intentional control. The resulting optimal trajectory therefore reflects not only a preference for reaching the desired cognitive state, but also a preference for maintaining continuity in the process of cognitive reorientation. This extension is conceptually related to smoothness priors in active inference and regularized SB formulations. Whereas the minimal model treats u(t) as an instantaneous steering variable, the extended model treats the temporal evolution of the control itself as a relevant dynamical object. An intriguing consequence of this extension is that the pair $\left(\rho_{t},u_{t}\right)$ becomes the fundamental dynamical entity of the theory. In the minimal formulation, only the quantum cognitive state $\rho_{t}$ evolves in time. After introducing the smoothness regularization, however, the control process itself acquires dynamical significance. The theory thus describes a joint evolution of cognitive states and cognitive control, suggesting a possible bridge between QSOT, active inference, and higher-order cognitive dynamics. These directions of extension will be a future challenge.

## 5. Discussion

### 5.1. Scope and Aim of the Present Model

The present work should not be understood as claiming to explain a specific behavioral data set in its current form. Rather, its aim is to provide a general mathematical framework for describing temporally extended, context-dependent cognitive dynamics. Many successful quantum cognitive models [3,26] have been constructed to account for particular empirical phenomena such as order effects, disjunction effects, conjunction fallacies, or violations of the sure-thing principle. The present approach is complementary to such models. Instead of targeting a single behavioral anomaly, it asks how quantum cognitive states may evolve over time under contextual, thermodynamic, and goal-directed constraints. In this sense, the toy model presented here is intended as a proof of concept. It demonstrates how a cognitive trajectory can be selected by a variational principle combining QSOT, quantum-logical updating, SB-like minimal deformation, and free-energy minimization. Future work should apply this framework to concrete behavioral paradigms, such as sequential judgment tasks, framing-dependent decisions, belief revision, confirmation bias, and temporal Bell-type tests in cognition [27]. Such applications will be necessary for quantitatively assessing the empirical advantage of the proposed framework over classical dynamical models.

### 5.2. Cognitive Interpretation and Relation to the Free Energy Principle

The mathematical structure introduced in this work can indeed be described, at an abstract level, as a constrained temporal evolution of a quantum-like state. Its cognitive interpretation arises from the operational meaning assigned to the state space, observables, projectors, and control field. In the intended application, the evolving density matrix represents an agent’s state over cognitive alternatives, such as competing beliefs, hypotheses, decisions, or action tendencies. The projection operators represent context-dependent questions, interpretive frames, or target propositions, while the control field represents endogenous modulation such as attention, deliberative effort, or task-directed reorientation. In this sense, the present model is not a physical quantum model of the brain, but a quantum-like model of temporally extended cognitive dynamics in the established sense of quantum cognition.

The terminology used in the model should therefore be understood in this operational sense. In particular, $H_{\mathrm{cog}}$ may be more precisely described as a cognitive preference Hamiltonian. It is not a microscopic Hamiltonian of neural tissue, but a Hermitian operator that defines a relative preference or stability structure over cognitive alternatives. In the present two-state example, the parameter Δ specifies the relative stability of one cognitive alternative over the other. Similarly, the contextual projector $\Pi_{\mathrm{ctx}}\left(t\right)$ should not be interpreted as a physical measurement apparatus, but as a time-dependent cognitive frame that changes the effective logical decomposition of the state space.

The relationship to the Free Energy Principle (FEP) should also be understood in a restricted and precise sense. The standard FEP and active inference are formulated in terms of an internal generative model of an external world, in which variational free energy is related to model evidence and sensory observations. The present model does not yet include such an explicit external world, sensory input, action, or generative model. Rather, it develops a free-energy-based variational structure for temporally extended quantum-like states. Thus, the proposed framework is inspired by the variational and uncertainty-reducing logic of the FEP, but it should not be regarded as a complete active-inference model.

From this viewpoint, the different terms in the action functional are not merely added for mathematical convenience. They correspond to distinct modeling roles: QSOT provides the temporally extended state representation, quantum-logical projectors represent context-dependent reframing, the SB-like term imposes minimal deviation from a spontaneous reference process, and the free-energy term represents a stability–uncertainty trade-off over cognitive alternatives. Constructing a full quantum-like generative model relating internal states to external causes and observations remains an important future direction.

### 5.3. Interpretation of the Temporal Structures Observed in Figure 1, Figure 2, Figure 3 and Figure 4

A notable feature of the numerical results above is the appearance of characteristic structures around t≈0, t≈ 5–6, and t≈8. These structures are consistently observed across multiple observables, including the terminal proposition trajectory, contextual projection probabilities, free-energy evolution, and the control-related quantities.

The structure near t≈0 reflects the initial contextual adaptation process. At the beginning of the evolution, the cognitive state is prepared in an initial proposition that is generally misaligned with both the contextual projector $\Pi_{\mathrm{ctx}}\left(t\right)$ and the terminal proposition $\Pi_{T}$. Consequently, the early stage of the trajectory is dominated by context-induced logical restructuring through repeated non-selective Lüders updates. This region may therefore be interpreted as an initial contextualization phase.

The feature observed around t≈ 5–6 appears to have a different origin. At this time, the context angle α(t) approaches a value close to π/4, where the contextual projector becomes approximately balanced between the two basis propositions. Simultaneously, the contextual target function $m_{\mathrm{ctx}}\left(t\right)$ has already passed its maximum and begins to decrease. As a result, the optimized trajectory experiences a transition between two competing tendencies: contextual alignment and terminal-goal steering. The resulting structure therefore represents a crossover region in which contextual consistency and goal-directed control contribute with comparable strengths. Rather than being a numerical artifact, this feature reflects the intrinsic compromise encoded in the variational principle.

Another transition-like feature appears around t≈8. By this stage, the terminal constraint begins to dominate the optimization process. The trajectory has already acquired substantial contextual alignment, and further evolution is primarily devoted to approaching the target proposition $\Pi_{T}$. This region may therefore be interpreted as a goal-consolidation phase. In cognitive terms, the system shifts from context-sensitive adaptation to the stabilization of a future-oriented decision state.

Taken together, the structures observed in the numerical trajectories suggest that the proposed framework naturally decomposes cognitive evolution into distinct temporal regimes: an initial contextualization phase, an intermediate contextual-goal crossover, and a final goal-consolidation phase.

### 5.4. Why Does the Introduction of Control Produce Only Modest Improvements?

An interesting aspect of the present results is that the introduction of the cognitive control field u(t) does not produce a dramatic improvement relative to the uncontrolled reference process. At first sight, this may appear surprising because the variational principle explicitly optimizes the control trajectory. However, this observation is in fact consistent with the conceptual foundations of the proposed theory. The optimized trajectory is not intended to maximize terminal performance at any cost. Instead, it is obtained by minimizing a composite action functional that simultaneously incorporates control expenditure, thermodynamic free energy, contextual logical consistency, and proximity to the uncontrolled reference process. The SB component of the objective function plays a particularly important role. Because the optimization penalizes excessive deviations from the reference process *Q*, the resulting trajectory is encouraged to remain close to the natural dynamics whenever possible. Consequently, the optimized solution generally represents the smallest modification of the reference process capable of satisfying the contextual and terminal constraints. From this perspective, the relatively modest quantitative improvement should not be interpreted as a weakness of the model. Rather, it reflects the fundamental principle that cognition is viewed as a constrained steering problem rather than a pure optimization problem. The goal is not maximal control but minimal intervention. The resulting trajectory may therefore be interpreted as the least-action cognitive path connecting contextual conditions to a desired future proposition. This interpretation is closely related to the original philosophy of Schrödinger bridges, where the optimal process is the most probable trajectory compatible with prescribed boundary conditions. In the present framework, cognition is similarly described as the least-deforming trajectory compatible with contextual and logical requirements.

### 5.5. Future Directions and Perspectives

The present work should be regarded as a proof-of-concept realization of the proposed quantum cognition theory. Although the numerical demonstrations were intentionally restricted to a two-level toy model, the theoretical framework itself is not limited to binary propositions or low-dimensional systems. Several directions for future research appear particularly promising.

First, the present model employs a single cognitive qubit and therefore does not yet capture genuinely multipartite phenomena. Extending the framework to tensor-product Hilbert spaces would enable the study of entanglement, contextual correlations, and collective cognitive representations. Such extensions may provide a natural connection to quantum contextuality and non-classical probabilistic structures observed in cognitive science.

Second, the current SB term is implemented through a state-wise QRE surrogate. A more rigorous formulation would involve path-space quantum bridges and quantum stochastic processes. Developing such a formulation may establish a deeper mathematical connection between QSOT, quantum optimal transport, and quantum information geometry [22,28,29,30].

Third, the present contextual projector evolves according to a prescribed trajectory. In realistic cognitive systems, contexts themselves should emerge dynamically from interactions among internal representations, memory structures, and environmental inputs. A future theory may therefore treat context as a dynamical variable rather than an externally imposed quantity.

Finally, the framework provides a possible route toward a quantum-logical generalization of the FEP. In the classical formulation, inference is performed within a fixed probabilistic and logical structure. In contrast, the present theory allows the logical framework itself to evolve through time-dependent contextual projections. If developed further, this perspective may offer a unified description of cognition as a process of quantum-logical state evolution constrained by free-energy minimization and optimal transport principles. The long-term objective is therefore not merely the construction of another quantum-cognitive model, but the development of a general theory of context-dependent cognition in which logical structure, temporal evolution, and variational optimization are treated within a single mathematical framework.

## 6. Conclusions

The central proposal advanced in this article is that temporal aspects of cognition and decision making may be described as variational dynamics on temporally extended quantum-like states.

In this work, we have proposed a new theoretical framework for temporal quantum cognition by integrating the basic ideas of the Free Energy Principle (FEP), Quantum State Over Time (QSOT), Schrödinger-bridge (SB) theory, and quantum logic into a unified variational formulation. Unlike conventional formulations of the FEP, which describe inference as Bayesian updating of instantaneous probability distributions, the present theory regards cognition as the evolution of temporally extended quantum states constrained simultaneously by thermodynamic stability, contextual logical consistency, and future-oriented goals.

The central hypothesis of the proposed theory is that cognition is not simply a sequence of isolated mental states but a temporally coherent quantum process. In this picture, a QSOT represents a unified cognitive object extending across past, present, and future, while its evolution is selected through an SB-type minimum-action principle. Context-dependent non-selective Lüders projections continuously reshape the underlying logical structure, allowing the logical framework itself to evolve during cognition rather than remaining fixed throughout inference.

The numerical simulations performed for a minimal two-state model illustrate how contextual constraints, free-energy minimization, and cognitive control collectively determine an optimal temporal trajectory. Although intentionally simple, the model already reproduces several characteristic features, including distinct phases of contextual adaptation, contextual-goal competition, and terminal goal consolidation. These results suggest that temporal organization itself may represent an essential aspect of cognitive dynamics.

The present work should be regarded as a proof-of-concept rather than a complete theory. Several important extensions remain to be developed. These include rigorous path-space quantum SB beyond the present path-integrated QRE approximation, higher-dimensional and multipartite Hilbert spaces capable of describing contextual entanglement, dynamically generated cognitive contexts, and applications to experimentally studied phenomena in quantum cognition and decision making.

More broadly, the proposed framework suggests a conceptual shift in our understanding of cognition. Rather than viewing cognition as inference performed on instantaneous mental states, it may be more appropriate to regard it as the optimal evolution of temporally extended quantum-logical structures under informational, thermodynamic, and contextual constraints. If this perspective proves fruitful, the present theory may provide a first step toward a unified mathematical description of cognition, decision making, and consciousness based on temporal quantum information. Recent quantum-like approaches to consciousness and qualia have also emphasized the usefulness of quantum-theoretical structures for describing measurement-dependent and context-dependent aspects of conscious experience [31]. The present framework is compatible with this broad direction, but it does not attempt to solve the hard problem of consciousness.

The classical FEP describes cognition as Bayesian inference over probability distributions. The present theory proposes a different viewpoint: cognition is the optimal evolution of temporally extended quantum-logical states. In this sense, the present dynamical theory on cognition may be regarded as a quantum-logical, free-energy-based variational framework inspired by the FEP.

## Data Availability

The data that support the findings of this study are available from the corresponding author upon reasonable request.

## Appendix A. Quantum Relative Entropy and Free-Energy Functionals

In the present framework the quantum relative entropy (QRE) between two density matrices ρ and σ is

(A1) $$D_{QRE}\left(\rho ∥\sigma \right)=\mathrm{Tr}\left(\rho \log\rho -\rho \log\sigma \right),$$

provided that supp(ρ)⊆supp(σ). This suggests a natural quantum counterpart of the variational free energy:

(A2) $$F\left(\rho ;\sigma \right)\equiv D_{QRE}\left(\rho ∥\sigma \right).$$

If ρ and σ commute and are diagonal in the same basis, F(ρ;σ) reduces to the Kullback–Leibler (KL) divergence $D_{\mathrm{KL}}$ between their eigenvalue distributions. Non-commutativity thus encodes genuinely quantum contributions to free energy.

From time-dependent density matrix ρ(t), the internal energy E(t), von Neumann entropy S(t) and Helmholtz free energy F(t)=E(t)−TS(t) are evaluated. The present formulation deliberately adopts an information-theoretic interpretation of von Neumann entropy. Although the mathematical structure resembles thermodynamic free energy, its role here is to quantify the trade-off between cognitive stability and cognitive uncertainty in a variational inference framework.

It is then found [13] that F(t) decreases toward an equilibrium value $F_{\mathrm{eq}}$, while S(t) often exhibits a non-trivial decrease, indicating the emergence of order (i.e., negentropy) [24,25] in the quantum decision process. This can be expressed by the inequality:

(A3) $$F\left(t\right)-F_{\mathrm{eq}}=\frac{1}{\beta }\;\mathrm{Tr}\left[\rho \left(t\right)(\log\rho \left(t\right)-\log\rho_{\mathrm{eq}})\right]\geq 0,$$

where $\rho_{\mathrm{eq}}\propto e^{-\beta H_{S}}$ refers to the density matrix in the equilibrium state. This expression is precisely the quantum relative entropy $D_{QRE}\left(\rho \left(t\right)∥\rho_{\mathrm{eq}}\right)$ and therefore coincides with the quantum variational free energy F(ρ(t);σ) with $\sigma =\rho_{\mathrm{eq}}$, where the difference from the reference part is considered. In other words, the dynamical free energy is a concrete realization of the quantum FEP functional $F\left(\rho ;\sigma \right)=D_{QRE}\left(\rho ∥\sigma \right)$ with the belief state ρ(t) and a Gibbs-type generative model σ, which corresponds to the variational posterior q(s) and the Bayesian inference p(s|o) in the classical FEP [1,2], respectively, where *s* and *o* refer to hidden states and observations.
